# McFarland Standards-Based Spectrophotometry Method for Calculating Approximate Multiplicity of Infection for an Obligate Intracellular Bacterium *Anaplasma phagocytophilum*

**DOI:** 10.3390/microorganisms13030662

**Published:** 2025-03-14

**Authors:** P. P. Mahesh, Jaydeep Kolape, Hameeda Sultana, Girish Neelakanta

**Affiliations:** 1 Department of Biomedical and Diagnostic Sciences, College of Veterinary Medicine, University of Tennessee, Knoxville, TN 37996, USA; mpp@utk.edu (P.P.M.); hsultana@utk.edu (H.S.); 2 Advanced Microscopy and Imaging Center, College of Arts and Sciences, University of Tennessee, Knoxville, TN 37996, USA

**Keywords:** human anaplasmosis, *Anaplasma phagocytophilum*, *Ixodes scapularis*, McFarland standard, optical density, spectrophotometry, multiplicity of infection

## Abstract

*Anaplasma phagocytophilum* is an obligate intracellular Gram-negative bacterium that causes human granulocytic anaplasmosis. Assessing the number of these bacteria is important for in vitro and in vivo infection studies. Colony count is used to set references for the multiplicity of infections in the case of culturable bacteria. However, the number of bacteria present inside the host cells, in which the bacteria are maintained, can be considered in the case of obligate intracellular bacteria. McFarland standards are a series of turbidity-based standards used to visually assess the approximate number of culturable bacteria. The turbidity of each standard can be related to their respective absorbances or optical densities (ODs). In this study, we describe a simple method to assess the approximate number of *A. phagocytophilum* based on McFarland standards. The ODs of cell-free crude extracts of *A. phagocytophilum* were used to assess the approximate number of bacteria while considering that the cell debris also contributes to the ODs. The consistency of this method was also tested using the bacterial cultures grown at different times. In summary, we provide a simple method to estimate the number of obligate intracellular bacteria for use in in vitro infection studies.

## 1. Introduction

*Anaplasma phagocytophilum* is the causative agent of human granulocytic anaplasmosis (HGA) formerly known as human granulocytic ehrlichiosis (HGE) [1,2]. The bacterium was first identified in the upper Midwest of the United States of America (USA) in 1994, and later in several other states of the USA [3,4]. *Anaplasma phagocytophilum* is also a multi-host pathogen that was detected in various animals worldwide [5,6,7]. In humans, the disease manifestations include fever, thrombocytopenia, malaise, myalgia, leukopenia, headache, and increased serum aminotransferase liver enzymes [8]. In the United States, *A. phagocytophilum* is primarily transmitted by *Ixodes scapularis* black-legged ticks in the Northeastern and Midwestern parts and *Ixodes pacificus* ticks in the Western part [9,10]. In ticks, this bacterium colonizes the salivary glands and is transstadially maintained in different developmental life stages [2,11,12].

*Anaplasma phagocytophilum* is an obligate intracellular Gram-negative bacterium that primarily infects and colonizes neutrophils in humans [1,2]. In neutrophils, *A. phagocytophilum* forms tiny clusters within the host-derived vacuoles called morulae [10,13]. *Anaplasma phagocytophilum* divides by binary fission within these morulae [10,13]. There are two forms of *A. phagocytophilum*, a larger reticulate (RC) form and a smaller dense core (DC) form [10,13,14,15]. In mammalian cells, only DC forms are shown to bind and enter the cells [10,13,15]. However, in tick cells, both RC and DC forms have been shown to bind and enter cells [14]. Upon bacterial entry into host cells and within 12 h post infection, the DC form converts to the reticulate form inside the morulae [10,13,14,15]. By 24 h post infection, morulae with numerous RC forms are observed [10,13,14,15]. By 36 h post infection, morulae with both RC and DC forms are observed, and the latter form is ready to exit and infect the neighboring naïve cells [10,13,14,15].

Because *A. phagocytophilum* is small and multiplies as RC and DC forms within a morula, it is impractical to accurately count the number of bacteria. The quantification of *A. phagocytophilum* by competitive PCR and electron microscopy have been reported [15,16]. In addition, the estimation of *A. phagocytophilum* based on the number of infected cells, the average number of morulae, the average number of bacteria within morulae, and the percentage of host cell-free bacteria recovered are also reported [17,18]. Recently, we reported the use of nCS1 analysis to count the number of *A. phagocytophilum* [19]. There are limitations on the development of methods to accurately count individual *A. phagocytophilum* bacterium. These could be due to the difficulty in controlling the number of morulae within the cell and controlling the number of RC or DC forms within each morula. In addition, it would be an impractical task to achieve these numbers by counting all infected cells individually and every time. To estimate *A. phagocytophilum*-containing morulae/cell or RC and DC forms/morula, microscopes such as electron microscopes must be used. Therefore, the development of cost-effective methods could facilitate an easy estimation of these bacteria. McFarland turbidity standards are used to visually assess the turbidity of a bacterial suspension by comparing it with a particular standard suspension [20]. A series of turbidity standards are prepared by mixing given volumes of 1% sulfuric acid and 1% barium chloride, resulting in barium sulfate suspensions [20,21]. The standards made of latex particles are also commercially available [22]. In this article, we report a simple method to estimate the number of *A. phagocytophilum* using a McFarland standard and spectrophotometry.

## 2. Materials and Methods

### 2.1. Bacterial Isolates, Cell Lines, and Infection

*The Anaplasma phagocytophilum* strain NCH-1 and tick cell line ISE6 was obtained from BEI Resources (Manassas, VA, USA). The HL-60 cell line and human endothelial cell line EA. Hy926 were obtained from ATCC, USA. The mCherry-*A. phagocytophilum* isolate was a kind gift from Dr. Ulrike Munderloh, University of Minnesota, MN, USA. Tick cell line ISE 6 was maintained in LI5B300 medium as described in our previous studies [23,24,25]. The EA.hy926 cell line was maintained in DMEM medium with 10% FBS. ISE6 cells and EA.hy926 cells were seeded 1 day prior to infection, and HL-60 cells were seeded on the same day of infection. For in vitro infections, *A. phagocytophilum* was isolated from HL-60 cultures maintained in IMDM medium with 10% FBS. Host cell-free *A. phagocytophilum* was isolated as described previously [23,26]. Briefly, 3 mL of *A. phagocytophilum*-infected HL-60 cells were centrifuged at 3000× *g* for 10 min. Cell pellets were resuspended in 3 mL of 1× PBS and incubated in −80 °C freezer for 10 min to allow an increased lysis of cells. Cells were passed through a 27-gauge syringe 6–8 times to release the bacteria. The cell debris was pelleted by centrifugation at 270× *g* for 3 min, and the supernatant was collected.

### 2.2. Preparation of McFarland Standards

McFarland standards were prepared as described [20]. The 1% sulfuric acid (Sigma, St. Louis, MO, USA) and 1% barium chloride (Sigma, St. Louis, MO, USA) solutions were prepared in ultrapure water and mixed accordingly to achieve different standards from 0.5 to 10. Barium sulfate precipitate was mixed thoroughly before measuring OD_600_ with a pathlength correction in Cytation 7 reader (BioTek, Shoreline, WA, USA).

### 2.3. DNA Extraction and Quantitative Real-Time PCR (QRT-PCR)

In total, 2 × 10^5^ of either tick cells, endothelial cells, or HL-60 cells were infected at various MOIs, and after 24 h p.i., cells were washed in 1X PBS. DNA was extracted using the DNeasy blood and tissue extraction kit (QIAGEN, Germantown, MD, USA). QRT-PCR was performed as described in our previous publications [23,26]. *Anaplasma phagocytophilum* loads in tick cells, endothelial cells, or HL-60 cells were measured by taking the ratios of the absolute amount of bacterial *p44* gene copies to that of 5.8S rRNA gene copies in tick cells or actin gene copies in the EA.hy926 and HL-60 cell lines. Standard curves for QRT-PCR for the *p44* gene fragment were prepared starting from 1 ng to 0.00001 ng/µL.

### 2.4. Fluorescence Imaging

In total, 3 × 10^4^ tick cells or endothelial cells were plated in a black transparent-bottom 96-well plate (Griener Bio One, ThermoFisherScientific, Waltham, MA, USA). After 24 h, cells were infected with mCherry-*A. phagocytophilum* at various MOIs. After 24 h p.i., the bright-field and mCherry fluorescence images of these cells were taken with a 40× magnification objective in the Cytation 7 imaging system (BioTek, Shoreline, WA, USA).

### 2.5. Transmission Electron Microscopy (TEM)

The TEM of *A. phagocytophilum* HL-60 cells was performed by using JEOL 1400 FLASH TEM (JEOL USA, Peabody, MA, USA) at 120 kV and as previously reported [15]. In total, 2 × 10^6^ HL-60 cells were infected with NCH-1, and after 5 days post infection, the cells were pelleted at 300× *g* and washed once in 0.1 M Sorenson phosphate buffer (SPB), at a pH of 7.4, to remove any media and fixed in 3.5% glutaraldehyde made in 0.1 M SPB for 1 h at room temperature under shaking. Cell pellets were subjected to four 15 min washes in 0.1 M SPB with 4% sucrose and stained with 1% Osmium tetroxide (OsO4) in 0.1 M SPB for 1 h at 4 °C. Samples were washed five times in 0.1 M SPB with 4% sucrose for 3 min each, followed by dehydration in a graded series of 30, 50, 70, 80, 90, and 100% ethanol for 5 min each. Next, propylene oxide (PO) was used to wash each sample twice for 5 min each, after which a 1:1 mixture of PO and epoxy resin was added. Samples were left overnight at room temperature. The next day, the PO–epoxy resin mixture was removed, and samples were incubated in epoxy resin for 5 h at room temperature. Samples were centrifuged, the resin was removed, fresh resin was added, and samples were allowed to harden at 60 °C for 48 h. Samples were thinly sectioned using Leica UC7 ultramicrotome (Leica Microsystems, Wetzlar, Germany), poststained with uranyl acetate and lead citrate, and examined under a transmission electron microscope.

### 2.6. Statistics

All the data sets were statistically analyzed using GraphPad Prism 9 software (www.graphpad.com). All data sets were initially tested for normal distribution by the Shapiro–Wilk normality test. For data sets with equal variances, the unpaired *t* test or one way ANOVA was used for a comparison of means; otherwise, the unpaired *t*-test or ANOVA with Welch correction were used. *p* values of <0.05 were considered significant.

## 3. Results

### 3.1. Preparation of McFarland Standards

**Figure 1:**
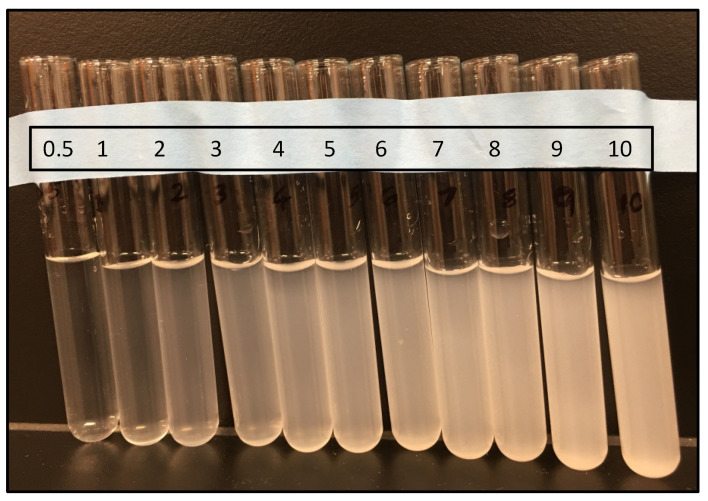
Preparation of McFarland standards. Image showing test tubes containing 1% barium chloride and 1% sulfuric acid that were mixed in proportions as shown in Table 1 to obtain a series of standards from 0.5 to 10. The turbidity of the solution increases from 0.5 to 10 in serial order of the standards. Picture is not to scale.

We first prepared McFarland standards using 1% each of barium chloride and sulfuric acid in ultrapure water. Eleven standards denoted as 0.5 to 10, in order of increasing turbidity, were made by mixing given volumes of 1% barium chloride and 1% sulfuric acid (Figure 1 and Table 1). The formation of barium sulfate precipitate was visible in all tubes resulting in an increasing order of turbidity from the standard 0.5 to 10 (Figure 1). The approximate number of bacteria/mL of the bacterial suspension corresponding to each standard is shown in Table 1. Each standard suspension was thoroughly mixed and a 200 µL suspension was added into a 96-well plate along with water as blank. Absorbance or optical density at 600 nm (OD_600nm_) was measured with pathlength correction, and the blank subtracted values are shown in Table 1.

### 3.2. OD Measurement of A. phagocytophilum in Crude Extracts Generated from HL-60 Cells

**Figure 2:**
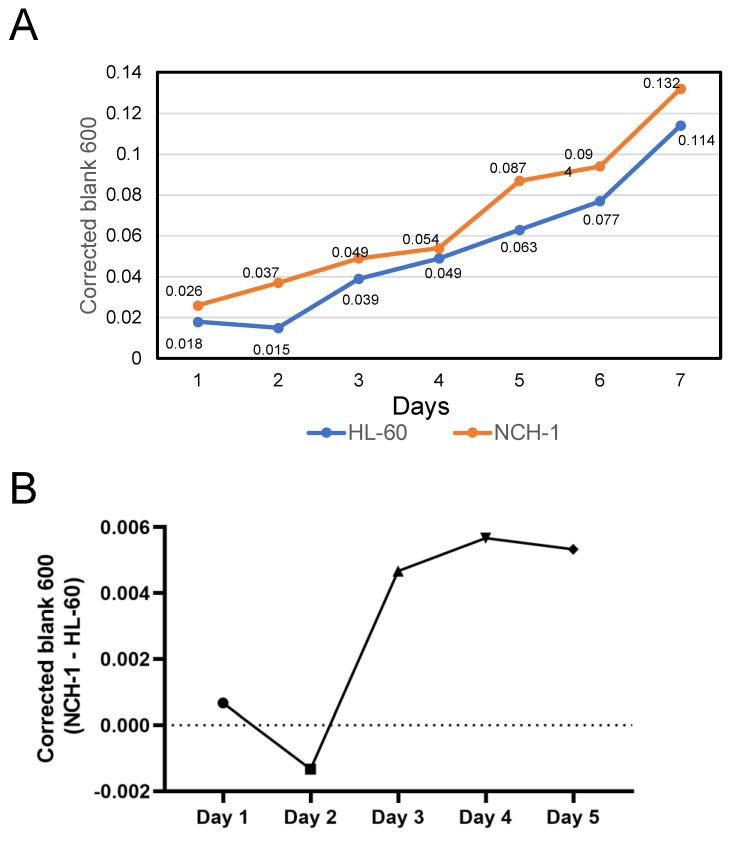
Optical density measurement of *A. phagocytophilum* culture crude extracts generated from infected HL-60 cells. (**A**) In total, 3 mL of 1.8 × 10^6^/mL of *A. phagocytophilum*-infected HL-60 cells and 3 mL of uninfected HL-60 cells (2.7 × 10^6^/mL) were mixed and grown for 7 days. The same volume of uninfected HL-60 cells was also inoculated and grown for 7 days. The optical densities (ODs) of the crude extracts of both cultures were plotted. (**B**) Equal numbers (1 × 10^6^) of HL-60 cells and *A. phagocytophilum*-infected HL-60 cells were mixed and grown for 8 days. The same number (2 × 10^6^) of uninfected HL-60 cells was also grown for 8 days. The OD differences obtained by subtracting uninfected HL-60 ODs from infected HL-60 ODs for days 1 to 5 are plotted.

The *Anaplasma phagocytophilum* strain, NCH-1, was maintained in HL-60 cells as described [23,26]. In the first experiment, a fresh culture of *A. phagocytophilum* was initiated by adding 3 mL (1.8 × 10^6^/mL) of infected HL-60 cells and 3 mL of uninfected HL-60 cells (2.7 × 10^6^/mL) and made up to 30 mL with IMDM medium containing 10% FBS. In addition, uninfected HL-60 cells were also grown by adding 3 mL of HL-60 inoculum (2.7 × 10^6^/mL) and making up to 30 mL with medium. From day 1 to day 7, 3 mL of each *A. phagocytophilum* culture was pelleted, suspended in 3 mL of 1× PBS, and the crude extract of the bacterium was prepared by passing through a 27.5-gauge syringe. The same procedure was followed in the case of HL-60 culture too. The OD_600_ of the crude extracts generated from *A. phagocytophilum*-infected or uninfected HL-60 cultures was measured in a spectrophotometer with a pathlength correction (Figure 2A). Increases in the OD_600_ values were noted over time in both crude extracts generated from *A. phagocytophilum*-infected or uninfected HL-60 cultures (Figure 2A).

In the second experiment, 2 × 10^6^ HL-60 cells were inoculated into 20 mL of medium as control. In another flask, 1 × 10^6^ uninfected HL-60 cells and 1 × 10^6^ infected HL-60 cells (*A. phagocytophilum* culture) were added to 20 mL of medium. Both cultures were grown in triplicates. Two milliliters of culture was drawn from each flask, pelleted and resuspended in 2 mL of 1X PBS every day for eight days, and proceeded for crude extract preparation, followed by the OD_600_ reading (Supplementary Figure S1). The mean OD_600_ values of crude extract prepared from *A. phagocytophilum*-infected HL-60 cultures were higher than the corresponding mean OD_600_ values of the crude extracts prepared from uninfected HL-60 cultures until day 5 post infection, except on day 2 (Supplementary Figure S1). However, the mean OD_600_ values of the crude extract prepared from *A. phagocytophilum*-infected HL-60 cultures were lower than the corresponding mean OD_600_ values of the crude extracts prepared from uninfected HL-60 cultures from days 6 to 8 p.i. (Supplementary Figure S1). Furthermore, the subtraction of the OD_600_ values of crude extracts prepared from uninfected HL-60 cells from the corresponding OD_600_ values of crude extracts prepared from *A. phagocytophilum*-infected HL-60 cells showed a logarithmic increase from day 2 to day 4 (Figure 2B). Collectively, these results show that the OD differences that resulted from subtracting the OD_600_ values of the crude extracts of the uninfected HL-60 cells from the OD_600_ values of the crude extracts of *A. phagocytophilum*-infected HL-60 cells are an approximate measure of the bacterial load in infected HL-60 cells.

### 3.3. Calculation of Approximate Number of Bacteria and Multiplicity of Infection (MOI) Using McFarland Standards

We randomly chose the McFarland standard 1 for assessing the number of bacteria. McFarland standard 1 corresponds to 3.0 × 10^8^/mL of bacteria approximately, and the same standard we prepared showed an OD_600_ equal to 0.261 (Table 1). Based on the data from the first experiment (Figure 2A), the OD_600_ values of the crude extracts generated from 7-day old uninfected HL-60 and *A. phagocytophilum*-infected HL-60 cells were noted to be 0.114 and 0.132, respectively. The difference in the OD_600_ values between these two samples is 0.018. The volume of the NCH-1 culture needed to infect 1 × 10^5^ cells at a multiplicity of infection (MOI) of 10 was calculated as shown below:

(1) $$\mathrm{Volume}\;\mathrm{in}\;\mu L\;=\frac{0.261\times 1000\times 1\times 10^{5}\times 10\;}{0.018\times 3\times 10^{8}}=48.33$$

The number of bacteria is directly proportional to the OD_600_ value of crude extracts generated from *A. phagocytophilum*-infected HL-60 cells. The volume needed to infect a particular number of cells at a particular MOI is inversely proportional to this OD_600_ value. If the same culture conditions are used every time, the following formula can be used to estimate the number of bacteria. The approximate volume of crude extract from 7-day-old *A. phagocytophilum*-infected HL-60 cell cultures with ‘‘X’’ OD needed to infect 1 × 10^5^ cells at a MOI of 10 is

(2) $$\mathrm{Volume}\;\mathrm{in}\;\mu L=\frac{0.132\times 48.33}{X}$$

### 3.4. Anaplasma Phagocytophilum Infection of Tick and Human Endothelial Cells Based on the MOI Determined Using the McFarland Method

**Figure 3:**
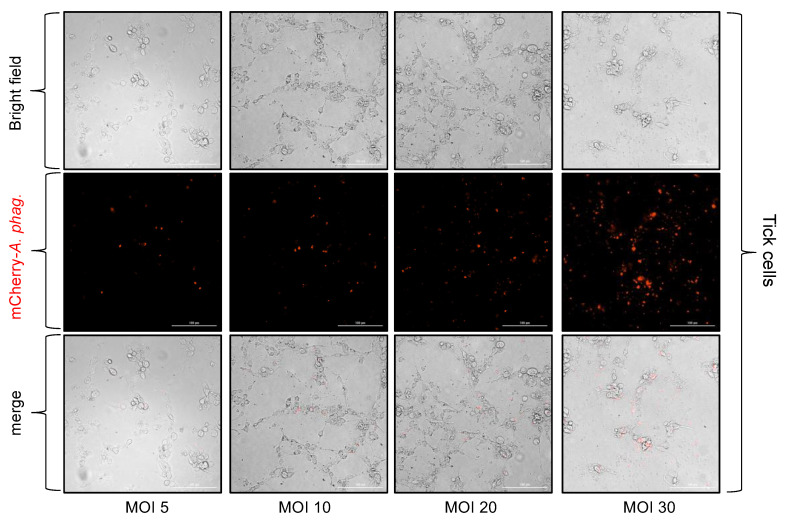
Infection of tick cells with mCherry-*A. phagocytophilum* at different MOIs determined from the McFarland standard. Phase contrast and fluorescent microscopic images showing mCherry-*A. phagocytophilum* infection (red staining) at different MOIs (5, 10, 20, and 30) in tick cells. After 24 h p.i., cells were processed for imaging using a fluorescent microscope with 40× objective. The scale bar indicates 100 μm.

**Figure 4:**
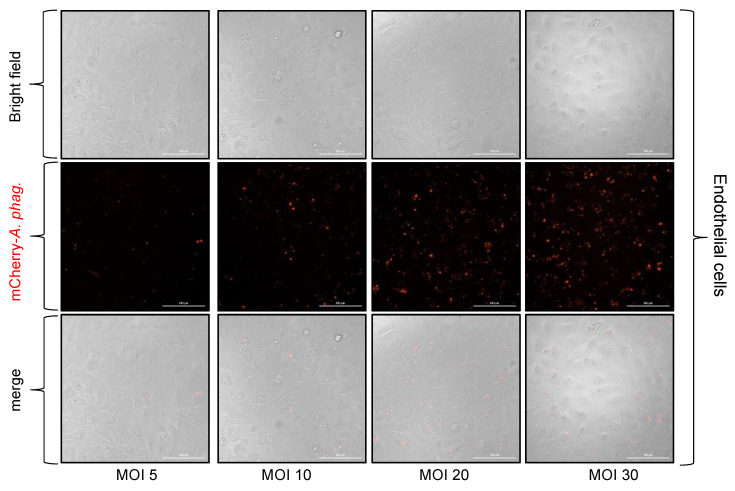
Infection of endothelial (EA.hy926) cells with mCherry-*A. phagocytophilum* at different MOIs determined by the McFarland standard. Phase contrast and fluorescent microscopic images showing mCherry-*A. phagocytophilum* infection (red staining) at different MOIs (5, 10, 20, and 30) in human endothelial cells (EA.hy926). After 24 h p.i., cells were processed for imaging using a fluorescent microscope with a 40× objective. The scale bar indicates 100 μm.

**Figure 5:**
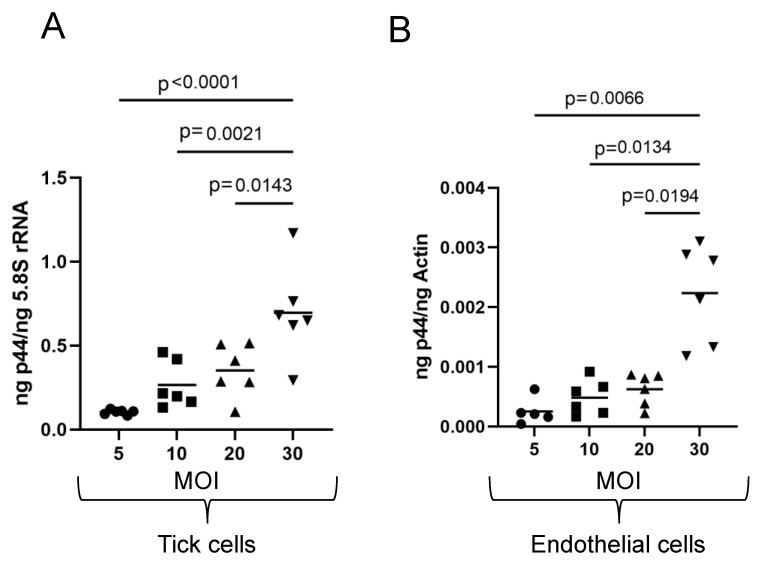
Quantitative measurement of bacterial loads in tick and endothelial cells upon infection with *A. phagocytophilum* at different MOIs determined by the McFarland standard. QRT-PCR analysis showing the levels of *A. phagocytophilum* in tick (**A**) or endothelial (**B**) cells upon infection at different MOIs (5, 10, 20, and 30) is shown. Bacterial levels (*A. phagocytophilum p44* gene) were normalized to host 5.8S rRNA levels or host beta-actin levels in DNA samples. Statistical analysis was performed with one-way ANOVA or ANOVA with Welch correction. Horizontal lines indicate mean. *p* < 0.05 is considered significant.

We then performed in vitro infection experiments with tick embryonic cell line, ISE6 (Figure 3), and the human endothelial cell line, EA.hy926 (Figure 4), and crude extracts generated from mCherry-expressing *A. phagocytophilum*-infected HL-60 cells. Both cells were plated at 3 × 10^4^ cells/well and infected with crude extracts generated from mCherry-expressing *A. phagocytophilum*-infected HL-60 cells at different MOIs (5, 10, 20, and 30) that were calculated based on the McFarland method. At 24 h p.i., both bright-field and fluorescent images were obtained. An increasing trend in the number of mCherry-expressing *A. phagocytophilum* was observed in both cells at 24 h p.i. infected with bacterial doses from 5 to 30 MOIs (Figure 3 and Figure 4). Furthermore, quantitative real-time PCR (QRT-PCR) analysis revealed significantly (*p* < 0.05) increased bacterial loads in tick cells (Figure 5A) and in endothelial cells (Figure 5B) infected with *A. phagocytophilum* at an MOI of 30 compared to the infection performed at MOIs of 5, 10, or 20 at 24 h p.i. Taken together, these results support the use of McFarland standards to determine an approximate *A. phagocytophilum* number for in vitro infection studies.

### 3.5. Infection of HL-60 Cells with A. phagocytophilum Based on the MOI Determined Using the McFarland Method

**Figure 6:**
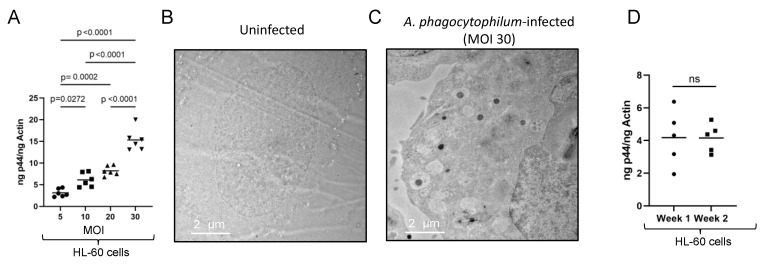
Quantitative measurement of bacterial loads in HL-60 cells upon infection with *A. phagocytophilum* at different MOIs determined by the McFarland standard. (**A**) QRT-PCR analysis showing levels of *A. phagocytophilum* HL-60 cells upon infection at different MOIs (5, 10, 20, and 30). Bacterial levels (*A. phagocytophilum p44* gene) were normalized to host beta-actin levels in DNA samples. Statistical analysis was performed with one-way ANOVA. Horizontal lines indicate mean. *p* < 0.05 is considered significant. (**B,C**) Representative TEM images of uninfected or *A. phagocytophilum*-infected HL-60 cells at five days p.i. are shown. HL-60 cells were infected at an MOI of 30 as determined by the McFarland method. Scale bars indicate 2 µm. (**D**) QRT-PCR showing *A. phagocytophilum* loads in HL-60 cells from two consecutive weeks. HL-60 cells were infected at an MOI of 10 in both weeks. The unpaired *t* test was used to compare the means. *p* < 0.05 is considered significant. ns means non significant.

We then determined bacterial loads in HL-60 cells infected with *A. phagocytophilum* at different MOI doses (5, 10, 20, and 30). QRT-PCR results showed significantly (*p* < 0.05) increased bacterial loads in HL-60 cells infected at an MOI of 30 compared to the infection performed at MOIs of 5, 10, or 20 (Figure 6A). In addition, significantly (*p* < 0.05) increased bacterial loads were evident in HL-60 cells infected at MOIs of 10 or 20 when compared to the infection performed at an MOI of 5 at 24 h p.i. (Figure 6A). To further validate this method, we performed transmission electron microscopy (TEM) studies. *Anaplasma phagocytophilum* culture was grown with the same proportion of HL-60 culture as described in the first experiment (Figure 2A). We plated 2 × 10^6^ HL-60 cells in duplicates. One well was infected at an MOI of 30 based on the McFarland method and the other well was kept as a control. Since the percentage of *A. phagocytophilum*-infected HL-60 cells is known to be very low at 24 h p.i. [15], these infected HL-60 cells were maintained for 5 days. The volume of crude extract needed for infection was calculated based on the equations given earlier. After 5 days of post infection, the cells were pelleted, washed, and processed for TEM studies (Figure 6B,C). As expected, we did not see any *A. phagocytophilum*-containing vacuoles in uninfected HL-60 control (Figure 6B). However, we noted the presence of multiple *A. phagocytophilum*-containing vacuoles in infected HL-60 cells (Figure 6C).

### 3.6. Consistency Noted with the Use of the McFarland Method in In Vitro Infection Studies with A. phagocytophilum

*A. phagocytophilum*-infected growing HL-60 culture (day 7 p.i.) was used to isolate bacteria and to infect 2 × 10^5^ HL-60 cells. The MOI was calculated based on the McFarland method, and HL-60 cells were infected at an MOI of 10 (week 1). On the same day, the isolated bacteria were used to inoculate a fresh culture of HL-60 cells. After day 7 p.i. (week 2), another infection of HL-60 cells with an MOI of 10 was performed like in week 1. DNA was isolated after 24 h p.i. in both cases and processed for QRT-PCR analysis. The QRT-PCR analysis revealed no significant (*p* > 0.05) differences in the bacterial loads between the samples (Figure 6D). These results indicate consistency in the McFarland counting method for enumerating *A. phagocytophilum* numbers for in vitro infection studies.

## 4. Discussion

Different types of methods including competitive PCR, electron microscopy, and the determination of the number of infected cells/morulae/bacteria within each morula have been reported for the quantification of *A. phagocytophilum* [15,16,17,18]. In this study, we describe a turbidity or OD-based method to approximately determine the number of *A. phagocytophilum*. McFarland standards are a set of turbidity standards used to visually compare them with the turbidity of bacterial cultures and calculate an approximate number of bacteria. Here, the measurement of absorbance or the OD of cultures can also be opted instead of visual comparison. The calculation of the MOI based on OD is widely followed in the case of culturable bacteria such as *Mycobacteria* [27,28,29]. The OD-based calculation of MOI is an easy and convenient method, even though it would give an approximate account of the number of bacteria. Based on this method, OD measurement is directly proportional to the approximate number of bacteria. The measurement of ODs from high-turbid cultures may yield false measurements due to the excessive scattering of light [30]. Therefore, bacterial cultures with higher turbidity must be diluted for the ease of OD measurements. We picked McFarland standard 1 to assess the number of bacteria from the corrected OD_600_ of the NCH-1 culture due to its less turbid nature.

We used *A. phagocytophilum* cultures (from days 6 or 7 p.i.) for infection studies. Since a considerable amount of cell debris contributes to the OD values, the respective OD value of uninfected HL-60 cultures must be subtracted from the OD values of *A. phagocytophilum*-infected HL-60 culture to assess the approximate number of bacteria. The experimental conditions used in the second set of experiments (Supplementary Figure S1) cannot be used for calculating the OD difference, since it will give a negative value for day 6 and above. The observation of lower ODs in crude extracts prepared from days 6–8 p.i. of *A. phagocytophilum*-infected HL-60 cultures compared to uninfected HL-60 cultures could be due to the possibility of cell death caused by the multiplying bacteria. This observation may not correlate with the PCR performed on the crude extracts prepared from days 6–8 p.i. and could be a difficult factor to evaluate the accuracy of the McFarland method. Therefore, the first experimental plan was used in all experiments to calculate the OD difference. To obtain an approximate number of bacteria, the OD values from crude extracts generated from uninfected HL-60 OD must be subtracted from the OD values from the crude extracts generated from *A. phagocytophilum*-infected HL-60 cells. This would minimize the OD value obtained from the cell debris. In addition, an actively growing culture of bacteria should always be used for this analysis. We used a maximum of eight passages of the NCH-1 culture and initiated a fresh culture from the frozen stock after that.

We used the plate reader to measure the OD_600_ of the standards and samples and used a default pathlength correction method in the reader to match the readings that are supposed to be obtained by using a standard spectrophotometry cuvette. Since the pathlength correction method based on the absorbance of water at 977 nm is not completely accurate, slight variations are expected in the corrected OD_600_ values. We noted similar OD values when the same amounts of inoculums of both HL-60 and NCH-1 cultures were used in different experiments. Furthermore, if there is no considerable change in the maintenance of cultures, the volume of NCH-1 crude extract needed for infecting a particular number of cells at a particular MOI can be assessed using the corrected blank 600 value of a previous NCH-1 extract. This notion is supported by the results in Figure 6D, where consistency in the bacterial loads was observed upon *A. phagocytophilum* infection of the HL-60 cells at two different time points that were one week apart.

The correlation of linear increase in the bacterial staining in mCherry-*A*. *phagocytophilum*-infected human endothelial cells and tick cells (infected at different MOIs as determined by the McFarland method) with bacterial loads detected by QRT-PCR further supports the use of this method to approximately calculate *A. phagocytophilum* numbers for performing in vitro infection studies. Even though this method may not provide an accurate number of *A. phagocytophilum* in the extracts, the method can be improvised for consistency by considering some of these parameters: the OD measurements of the extracts upon the infection of cells with different doses of bacteria, the OD measurements of extracts generated from different cells, the use of actively growing cultures, the use of similar isolation methods, and the use of similar inoculation and culture conditions every time. In summary, we believe that the method we presented in this study is rapid, easy to follow, and useful to obtain an estimation on the *A. phagocytophilum* numbers for in vitro infection studies.

## Figures and Tables

**Table 1:** The McFarland standards. Table showing a series of McFarland standards from 0.5 to 10, equivalent number of bacteria/mL, and their respective corrected OD_600_ values.

McFarland Standard	1% Barium Chloride (mL)	1% Sulfuric Acid (mL)	Approximate Bacterial Number/mL	Corrected Blank 600
0.5	0.05	9.95	1.5 × 10^8^	0.118
1	0.10	9.90	3.0 × 10^8^	0.2615
2	0.20	9.80	6.0 × 10^8^	0.4745
3	0.30	9.70	9.0 × 10^8^	0.822
4	0.40	9.60	1.2 × 10^9^	1.1555
5	0.50	9.50	1.5 × 10^9^	1.4735
6	0.60	9.40	1.8 × 10^9^	2.039
7	0.70	9.30	2.1 × 10^9^	2.581
8	0.80	9.20	2.4 × 10^9^	3.3545
9	0.90	9.10	2.7 × 10^9^	4.188
10	1.00	9.00	3.0 × 10^9^	5.2365

## Data Availability

The original contributions presented in this study are included in the article/Supplementary Materials. Further inquiries can be directed to the corresponding author.

## Supplementary Materials

The following supporting information can be downloaded at: https://www.mdpi.com/article/10.3390/microorganisms13030662/s1, Figure S1: Optical density measurements of NCH-1 and HL-60 cultures grown with equal number of cells.
