# Supplementary material for: McFarland Standards-Based Spectrophotometry Method for Calculating Approximate Multiplicity of Infection for an Obligate Intracellular Bacterium Anaplasma phagocytophilum
Source: Microorganisms. 2025 Mar 14;13(3):662. doi: 10.3390/microorganisms13030662 (PMC11945594; doi:10.3390/microorganisms13030662)
Supplement: Supplementary file 1 [file microorganisms-13-00662-s001.zip › microorganisms-3478491-supplementary.pdf]

McFarland standards-based spectrophotometry method for calculating approximate multiplicity of infection for an obligate intracellular bacterium *Anaplasma phagocytophilum*

P P Mahesh [1], Jaydeep Kolape [2], Hameeda Sultana [1], and Girish Neelakanta [1, *]

Supplementary information

Supplementary Figure legends

Supplementary figure 1. Optical density measurements of NCH-1 and HL-60 cultures grown with equal number of cells. Equal number of cells (2×10^6) were seeded in two separate cultures, one with uninfected HL-60 cells alone, and another with uninfected HL-60 cells and NCH-1-infected HL-60 cells at 1:1 proportion. Both cultures in triplicates were grown for 8 days and mean ODs of the crude extracts were plotted.

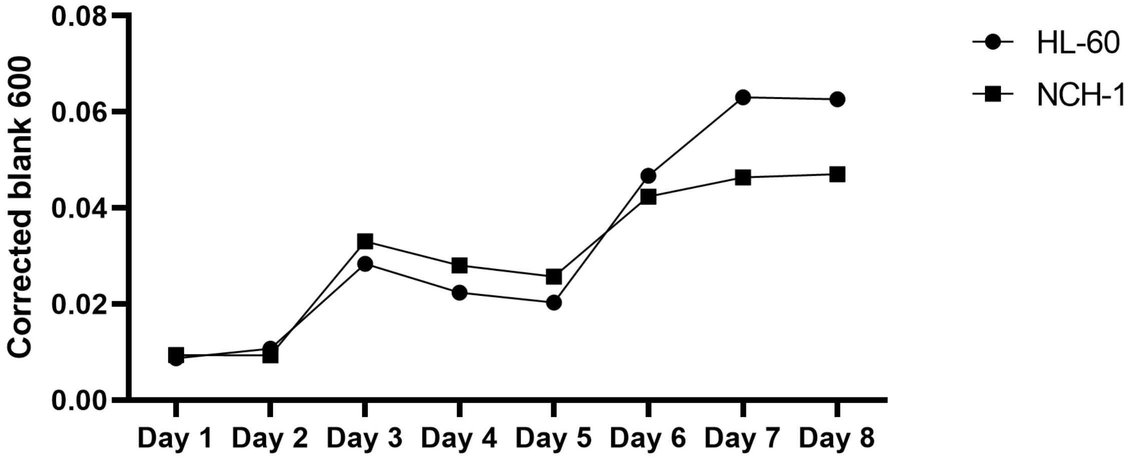

Supplementary figure 1
